# Vitamin D Status and Glycemic Control: Experience of the Biochemistry Laboratory, Mohammed VI University Hospital, Oujda, Morocco

**DOI:** 10.7759/cureus.101615

**Published:** 2026-01-15

**Authors:** Kholoud Krimi, Dounia El Moujtahide, El-Houcine Sebbar, Mohammed Choukri

**Affiliations:** 1 Laboratory of Biochemistry, Central Laboratory Department, Mohammed VI University Hospital, Faculty of Medicine, Mohammed First University, Oujda, MAR

**Keywords:** 25-hydroxyvitamin d, diabetes mellitus, glycemic control, hba1c, hypovitaminosis d, vitamin d

## Abstract

Background

Vitamin D is best known for its role in calcium and phosphate homeostasis, but it may also influence metabolic function. It has been suggested that vitamin D may be linked to glucose regulation. Accordingly, vitamin D status has attracted considerable interest as a potential determinant of glycemic control in diabetes. Although multiple studies have suggested that lower serum 25(OH)D concentrations are associated with higher HbA1c, the overall evidence remains mixed.

Objective

This study aimed to evaluate the association between serum 25(OH)D and glycated hemoglobin (HbA1c) among hospitalized diabetic patients at Mohammed VI University Hospital, a tertiary care hospital in Oujda, Morocco, and to assess whether hypovitaminosis D is linked to uncontrolled diabetes.

Methods

We conducted a retrospective, cross-sectional study in the Biochemistry Department over a 12-month period. Hospitalized patients with known type 1 diabetes (T1D) or type 2 diabetes (T2D) who had 25(OH)D and HbA1c measured during the same admission were included; only the first paired measurements were retained. Patients with missing data or conditions likely to bias vitamin D or HbA1c interpretation were excluded. Serum 25(OH)D and HbA1c were measured using routine laboratory methods. Vitamin D status was categorized as <10 ng/mL, 10-30 ng/mL, and >30 ng/mL. Glycemic control was defined as controlled versus uncontrolled using a prespecified HbA1c threshold.

Results

A total of 100 patients were included (45 men, 55 women; mean age 53 years; range 8-85), including 79 with T2D and 21 with T1D. The mean 25(OH)D was 20.84 ± 11.56 ng/mL, and the mean HbA1c was 7.15 ± 1.64%. The mean HbA1c decreased stepwise across vitamin D categories (8.90%, 7.01%, and 6.36%, respectively). The prevalence of uncontrolled diabetes was 80.0% (12/15) in the <10 ng/mL group, 34.9% (22/63) in the 10-30 ng/mL group, and 4.5% (1/22) in the >30 ng/mL group (p < 0.05). Serum vitamin D was inversely associated with HbA1c in continuous analysis (p < 0.05). When dichotomized, uncontrolled diabetes was more frequent with low vitamin D (≤30 ng/mL) than with normal vitamin D (>30 ng/mL) (43.6% vs. 4.5%; p < 0.05).

Conclusion

In this hospitalized diabetic cohort, lower serum 25(OH)D levels were significantly associated with higher HbA1c and a markedly greater prevalence of uncontrolled diabetes. While causality cannot be inferred from this retrospective design, hypovitaminosis D may identify patients at higher risk of glycemic disequilibrium, and further studies are needed to clarify clinical implications after accounting for key confounders.

## Introduction

Vitamin D has received growing attention in recent years in both research and the scientific literature. Beyond its established role in calcium and phosphate homeostasis, accumulating evidence suggests that vitamin D may exert a range of effects, potentially influencing the development and progression of several diseases, including diabetes [1]. This has led to increased interest in the possible contribution of vitamin D deficiency to the pathophysiology of diabetes [2]. In parallel, observational studies have reported a higher prevalence of type 2 diabetes (T2D) among individuals with low serum vitamin D levels [3]. Although vitamin D supplementation has been considered as a potential strategy for diabetes prevention, its use must be balanced against potential adverse effects, particularly hypercalcemia [4].

In this context, we conducted a retrospective study to evaluate the relationship between serum vitamin D and glycated hemoglobin (HbA1c) among diabetic patients hospitalized at Mohammed VI University Hospital, Oujda, Morocco, and to assess whether hypovitaminosis D is associated with poorer glycemic control. Despite the limitations inherent to our study design, we observed lower vitamin D levels among patients with uncontrolled diabetes.

## Materials and methods

Study design and setting

This retrospective, cross-sectional study was conducted in the Biochemistry Department of Mohammed VI University Hospital (Oujda, Morocco) over a 12-month period (October 1, 2022, to October 1, 2023). We included hospitalized patients with known diabetes mellitus (type 1 or type 2) who had serum 25-hydroxyvitamin D [25(OH)D] and HbA1c measured during the same hospital admission. For patients with repeated measurements, only the first paired results were retained.

Laboratory methods

Serum 25(OH)D was measured by chemiluminescent immunoassay on the Abbott Alinity c platform, and HbA1c was measured using the ADAMS TM-8180T analyzer.

Inclusion criteria

Eligible participants were hospitalized at Mohammed VI University Hospital during the study period, had a known diagnosis of diabetes mellitus (type 1 or type 2), and had both serum 25(OH)D and HbA1c measured during the same hospital stay.

Exclusion criteria

Patients were excluded if either 25(OH)D or HbA1c data were missing (n = 9) or if they had conditions likely to bias the interpretation of vitamin D status or HbA1c (n = 15), including advanced chronic kidney disease, severe liver failure, or recent vitamin D therapy prior to testing.

Data collection

Data were extracted from laboratory records and included demographic variables (age and sex), diabetes type, and paired 25(OH)D and HbA1c results obtained during the same admission. All data were anonymized prior to analysis, entered into Microsoft Excel, and checked for completeness and consistency.

Statistical analysis

Continuous variables were summarized as means, while categorical variables were presented as counts and percentages. Patients were categorized by vitamin D status (<10 ng/mL, 10-30 ng/mL, >30 ng/mL) and by glycemic control (controlled: HbA1c ≤ 7.5% vs. uncontrolled: HbA1c > 7.5% based on the prespecified HbA1c threshold). Group comparisons of HbA1c were performed using independent-samples t-tests (Welch correction when variances were unequal), and associations between categorical variables were assessed using the chi-square test. Statistical analyses were performed using MS Excel (Microsoft Corp., USA), and a p-value <0.05 was considered statistically significant. The sample size was determined by the number of eligible patients available during the study period.

## Results

A total of 100 patients were included: 45 men and 55 women, with a male-to-female ratio of 0.82. Ages ranged from eight to 85 years, with a mean age of 53 years. T2D was present in 79 patients and type 1 diabetes (T1D) in 21. Vitamin D status was categorized as Group A (<10 ng/mL), Group B (10-30 ng/mL), and Group C (>30 ng/mL). Glycemic control was defined as controlled (HbA1c ≤7.5%) versus uncontrolled (HbA1c >7.5%); 65 patients (65%) were controlled, and 35 (35%) were uncontrolled. The mean HbA1c decreased stepwise across vitamin D categories (8.90% in Group A, 7.01% in Group B, and 6.36% in Group C) (Table 1).

**Table 1:** Distribution of the vitamin D status and glycemic control

Category	n (%)
Vitamin D	
Deficient (<10)	15 (15.0)
Insufficient (10–30)	63 (63.0)
Normal (>30)	22 (22.0)
Glycemic control (HbA1c)	
Controlled (≤7.5%)	65 (65.0)
Uncontrolled (>7.5%)	35 (35.0)

Among the 100 patients included, serum vitamin D had a mean value of 20.84 ng/ml ± 11.56, ranging from 5.2 to 50.1 ng/ml. Mean HbA1c was 7.15 ± 1.64%, ranging from 4.2 to 11.8% (Table 2).

**Table 2:** Descriptive statistics of the study variables IQR, interquartile range

Variable	Mean	Median (IQR)	Range
Vatiamin D	20.84 ± 11.56	17.0 (12.0-27.0)	5.2-50.1
HbA1c (%)	7.15 ± 1.64	6.75 (6.0-8.03)	4.2-11.8

The mean HbA1c was higher (>7.5%) in Group A (8.90%) than in Group B (7.01%) and Group C (6.36%) based on the study definition of glycemic control. When stratified by the vitamin D status, HbA1c showed a graded pattern. The mean HbA1c was 8.90% in the vitamin D-deficient group, 7.01% in the insufficient group, and 6.36% in the normal group.

Consistently, the proportion of uncontrolled diabetes was 80.0% (12/15) among vitamin D-deficient patients, 34.9% (22/63) among vitamin D-insufficient patients, and decreased to 4.5% (1/22) among patients with normal vitamin D levels. Statistically, the association between vitamin D categories and glycemic control was significant (p = 0.000014) (Table 3).

**Table 3:** Cross-tabulation of the vitamin D status and glycemic control P-values were calculated using the chi-square test of independence.

Vitamin D status	Controlled (≤7.5%), n (%)	Uncontrolled (>7.5%), n (%)	p-value
Deficient (<10)	3 (20.0)	12 (80.0)	0.000014
Insufficient (10–30)	41 (65.1)	22 (34.9)	
Normal (>30)	21 (95.5)	1 (4.5)	

The patients were classified according to serum vitamin D into low vitamin D (≤30 ng/mL; n = 78) and normal vitamin D (>30 ng/mL; n = 22). Mean HbA1c was 7.38% in the low vitamin D group compared with 6.36% in the normal vitamin D group, and this difference was statistically significant, with a 95% CI (Welch t-test, p = 0.000069). The magnitude of this difference was moderate (Cohen’s d = 0.64), indicating a clinically meaningful separation between groups. Uncontrolled glycaemia (HbA1c > 7.5%) was also more frequent in the low vitamin D group (43.6%) than in the normal vitamin D group (4.5%), with a significant association (chi-square, p = 0.00070) (Table 4). Overall, higher vitamin D levels were associated with better glycaemic control in this cohort.

**Table 4:** HbA1c and glycaemic control by vitamin D group (≤30 vs. >30 ng/mL), with p-values from the Welch t-test (HbA1c mean) and chi-square test (controlled vs. uncontrolled).

Vitamin D group (ng/mL)	n	HbA1c mean (%)	SD (HbA1c)	Controlled (≤7.5%) n (%)	Uncontrolled (>7.5%) n (%)	p-value (HbA1c, Welch t-test)	p-value (controlled vs. uncontrolled, chi-square)	Cohen’s d
≤30	78	7.38	1.77	44 (56.4%)	34 (43.6%)	0.000069	0.00070	0.64
>30	22	6.36	0.65	21 (95.5%)	1 (4.5%)			

We used the Welch t-test to compare mean HbA1c between the two vitamin D groups (≤30 vs. >30) because HbA1c is a continuous variable and the two groups had unequal sample sizes and potentially unequal variances, making Welch’s test the more appropriate and robust choice. We used the chi-square test of independence to examine the association between categorical variables, vitamin D group, and glycaemic control status (controlled vs uncontrolled), because this test evaluates whether the proportions of controlled/uncontrolled patients differ across vitamin D categories.

## Discussion

Vitamin D has functions that extend far beyond calcium-phosphate homeostasis, including roles in immune regulation, associations with cancer risk and outcomes, and, more recently, investigation as a potential modulator of diabetes risk [2].

In our study, vitamin D status was significantly associated with glycemic control. Specifically, vitamin D deficiency was accompanied by higher glycated hemoglobin levels, suggesting that lower vitamin D levels may be linked to poorer glycemic balance in diabetic patients. This aligns with population-based data, including the National Health and Nutrition Examination Survey, which reported an inverse relationship between serum vitamin D concentrations and indices of glycemic control.

Vitamin D may be obtained through dietary sources of plant or animal origin and is also synthesized in the skin following ultraviolet exposure. Vitamin D₃ undergoes two sequential hydroxylations-first in the liver and then in the kidney-to generate the biologically active metabolite, 1,25-dihydroxyvitamin D [5]. Low vitamin D levels are frequently reported in individuals with type 2 diabetes mellitus (T2DM) and may contribute to metabolic disturbances, including β-cell dysfunction, insulin resistance, and pro-inflammatory states, which collectively can worsen glucose homeostasis. Pittas et al., 2023 [6]. Multiple studies have suggested an association between vitamin D status and the risk of T2D [6].

T2D is a multifactorial condition influenced by genetic predisposition, environmental factors, obesity, and sedentary lifestyle [2]. It is typically preceded by insulin resistance and impaired glucose tolerance [7]. Several observations support a potential link between vitamin D and T2D pathophysiology, including reports of worse glycemic control during winter compared with spring [8]. In addition, some studies have suggested that vitamin D supplementation may improve glucose regulation in patients with T2D [9].

In T1D, a dose-dependent protective association has also been proposed. Regular vitamin D supplementation early in life has been linked to a lower risk of developing T1D, as reported by Stene et al. [10], and childhood vitamin D deficiency has been consistently associated with a higher risk of T1D [11].

Supporting clinical observations have been reported across different populations. Alhussar et al. found that patients with T2D exhibit marked differences in vitamin D status, bone metabolism markers, and glycemic parameters [5]. In that study, vitamin D deficiency was more pronounced in the diabetic group, with lower serum 25(OH)D concentrations than in controls [5]. They also reported a significant inverse association between serum phosphate levels and markers of glycemic control, including HbA1c and fasting plasma glucose. In pediatrics, Savastio et al. reported that 25(OH)D deficiency is frequent among children with T1D and suggested that supplementation could potentially support glycemic control and insulin sensitivity, thereby improving disease management [12]. Similarly, Ahi et al. observed that higher HbA1c levels (≥ 8%) were associated with lower vitamin D levels, whereas lower HbA1c values (< 7.5%) were associated with higher serum vitamin D concentrations [13]. They also raised the possibility that lower vitamin D status may be linked to diabetic vascular complications, including diabetic retinopathy.

However, evidence from interventional trials remains mixed. In a multicenter, randomized, placebo-controlled trial by Pittas et al. [14], daily vitamin D₃ supplementation (4000 IU) in individuals at high risk for T2D, who were not selected based on vitamin D insufficiency, did not significantly reduce diabetes incidence compared with placebo over a median follow-up of 2.5 years [14]. In this context, our findings emphasize that vitamin D deficiency is associated with a higher likelihood of glycemic disequilibrium among diabetic patients compared with those in other vitamin D categories, although causal inference cannot be established from our study design.

The limitations of our study include the absence of adjustment for several potential confounders, such as BMI/obesity, seasonality, medications, outdoor activity and sun exposure, physical activity, and dietary habits. In addition, the single-center design and the inclusion of hospitalized patients may limit generalizability and introduce selection bias. Finally, we relied on a single marker (HbA1c) to assess glycemic control and did not incorporate contemporaneous serum glucose measurements in our analysis.

## Conclusions

This study suggests that vitamin D status may be clinically relevant when evaluating hospitalized patients with diabetes, as low vitamin D levels can accompany less favorable glycemic control and may help identify individuals who warrant closer metabolic assessment and optimization. Given the observational design, these findings should be interpreted as an association rather than evidence of a causal effect of vitamin D on glucose regulation. Prospective, multicenter studies, ideally randomized trials of standardized vitamin D repletion are needed to determine whether correcting vitamin D deficiency meaningfully improves glycemic outcomes in patients with diabetes.
